# An HIV Associated Plasmablastic Lymphoma With Spontaneous Tumor Lysis Syndrome

**DOI:** 10.7759/cureus.9625

**Published:** 2020-08-09

**Authors:** Ali F Al Sbihi, Paramveer Singh, Nouraldeen Manasrah, Emad Kandah, Joel Appel

**Affiliations:** 1 Internal Medicine, Detroit Medical Center - Sinai-Grace Hospital, Detroit, USA; 2 Internal Medicine, McLaren Flint, Flint, USA

**Keywords:** plasmablastic lymphoma, spontaneous tumor lysis syndrome, hiv lymphoma

## Abstract

Plasmablastic lymphoma (PBL) is a rare and aggressive B-cell Non-Hodgkin lymphoma (NHL) associated with immunocompromised states such as HIV. We present a case of PBL in an HIV patient presenting as spontaneous tumor lysis syndrome and discuss the clinical challenges hence encountered.

## Introduction

Plasmablastic lymphoma (PBL) is a B-cell lymphoma that was previously considered a subtype of terminally differentiated diffuse large B-cell lymphoma with plasmablastic differentiation; currently, it is recognized as a distinct entity of B-cell lymphoma [1]. It is often associated with immunosuppressive conditions such as the human immunodeficiency virus (HIV). In HIV patients, 2.6% of Non-Hodgkin's lymphoma (NHL) is found to be due to PBL [2]. Usually, it presents as an advanced stage (stage III or IV). Spontaneous tumor lysis syndrome is a rare complication of this rare lymphoma [3]. The aggressive nature of this tumor requires prompt diagnosis and treatment. In this report, we discuss a case of a 49-year-old man presenting with spontaneous tumor lysis syndrome secondary to rapidly dividing PBL.

## Case presentation

**Figure 1:**
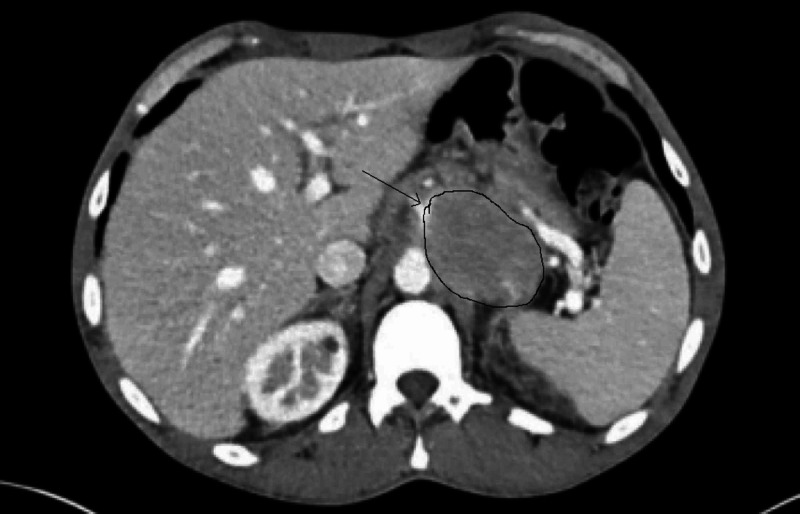
CT scan of the abdomen Contrast-enhanced CT abdomen demonstrating a mildly enhancing soft tissue mass in the lesser sac invading the splenic artery.

Our patient is a 49-year-old African-American man with the past medical history of gastritis and HIV since 2004; on anti-retroviral therapy (ART) of bictegravir, emtricitabine, and tenofovir alafenamide, he was compliant to his medications for the last seven years, and inconsistently before that, he had a CD4 count of 375 cells/Ul (normal: 500-1600 cells/Ul) and HIV viral load of 122 copies, the patient presented to the emergency department with the chief complaint of epigastric pain associated with persistent nausea, loss of appetite and episodic vomiting for few days. His symptoms had not resolved with proton pump inhibitors; he noticed weight loss and denied fever or night sweats. CT of the abdomen with contrast revealed 7.0*5.0*5.0 cm mildly enhancing soft tissue mass in the lesser sac invading the splenic artery (see Figure 1). CT-guided core needle biopsy of the mass was performed, and histopathological findings were consistent with the features of PBL (monomorphic diffuse lymphoid cells of plasmablastic morphology positive for CD38, CD138, MUM1 immunostains, and negative for CD20 and CD79a, Ki-67 was not checked). In situ hybridization for Epstein-Barr viral RNA (EBER) was negative. The patient improved clinically and was discharged to follow up as an outpatient with an oncologist for staging and treatment. On discharge, creatinine was 1.2 mg/dl, and hemoglobin was 11.6 gm/dl. Unfortunately, five days after discharge, the patient was rushed to the emergency department in a critical condition - he had profound hypotension (blood pressure: 71/55 mmHg) and acute kidney injury (creatinine: 4.11 mg/dl). He was admitted to the hospital where further workup revealed findings consistent with tumor lysis syndrome (uric acid: 14.1 mg/dl, phosphorous: 11.6 mg/dl, K: 6.2 mMol/L, calcium: 8.8 mg/dl, hemoglobin: 5.3 gm/dl, hematocrit: 15.4%, lactate dehydrogenase (LDH): 4704 U/Liter, haptoglobin: <30 mg/dl, corrected reticulocyte count: 1.8%, reticulocyte count: 69.300 cells/mm3), and negative coombs test. It was determined that the patient had Cairo-Bishop criteria (3/4 laboratory and grade II clinical). The patient received intravenous fluid resuscitation, dexamethasone 4 mg twice daily, rasburicase, allopurinol, and other supportive measures as a blood transfusion. The patient continued to have worsening kidney function and hyperkalemia resistant to medical therapy, so hemodialysis was to be done but was refused by the family who changed the code status to comfort measures. The patient expired one week after the presentation, and two weeks after the PBL diagnosis was made. Unfortunately, time did not allow for fluorescence in situ hybridization (FISH), comprehensive staging, or the initiation of chemotherapy.

Immunohistochemistry lab reported the following results (Fugures 2-3): the neoplastic cells were positive for CD45, CD4, CD138 (syndecan-1), MUM-1, and c-myc; whereas cells were negative for cytokeratin AE1/AE3, CD3, CD5, CD79a, CD19, CD20, CD8, CD22, CD10, BCL-6, BCL-2, CD30, CD56, and ALK-1.

**Figure 2:**
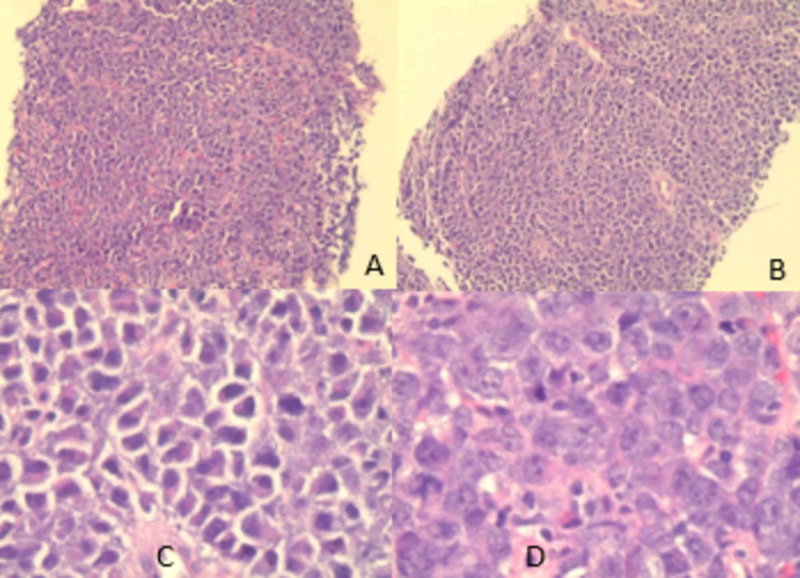
CT scan guided biopsy results Images A and B are low power view. Image C is a high power view showing plasmacytoid features with hexcentric nuclei and paranuclear hofs. Image D shows prominent nucleoli.

**Figure 3:**
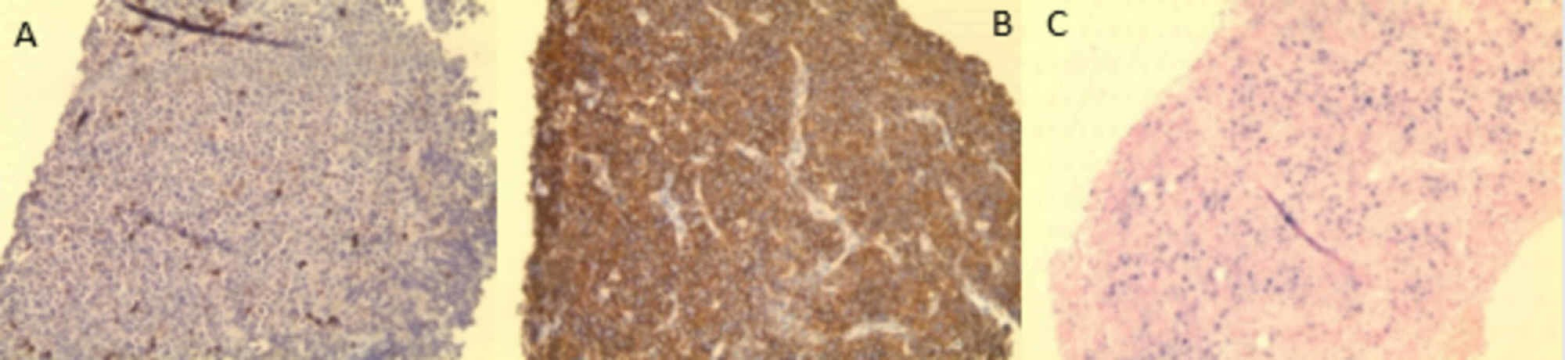
Immunohistochemistry lab results Image A shows cells which are negative for CD79a. Image B shows positive CD138 and image C shows negative Epstein-Barr virus (EBV) stain.

## Discussion

Plasmablastic lymphoma is a rare B-cell NHL, which is characterized by its aggressive presentations in HIV patients. Historically, it is associated with HIV diagnosis; some cases were reported in transplant patients or steroid therapy for autoimmune diseases [4]. Many cases were reported in HIV negative patients. The majority of cases are EBV positive. Male:female ratio is around 3:1 [5]. The oral cavity represents the most common location followed by other locations such as the gastrointestinal tract, lymph nodes, visceral cranium, cervix, thorax, skin, and retroperitoneum [6-10]. The prognosis of patients with PBL is generally poor with a median overall survival of 6-19 months, and there are no clear cut differences between HIV-positive and HIV-negative patients based on a meta-analysis of 277 patients [11, 12]. It was reported in 122 cases review of literature that the median overall survival time for the whole group was 14 months, with a five-year survival rate of 31%. Variation mostly depends on stage, chemotherapy response, and antiretroviral therapy [13]. Prospective clinical trials for treatment options are lacking; therapy is considered based on available case reports and small retrospective case series.

Tumor lysis syndrome (TLS) can be the first manifestation of an underlying malignancy; it can rarely occur in rapidly dividing leukemia, lymphoma, or solid tumors. TLS is a potentially fatal oncologic emergency, and it is particularly important that clinicians recognize the laboratory (uric acid, potassium, calcium, and phosphorus) and clinical findings that suggest the diagnosis. TLS can lead to many complications, such as acute kidney injury, arrhythmias, seizures, and rarely liver damage.

TLS is more commonly seen after treatment of hematological malignancies such as acute leukemia and NHL; incidence is generally decreased with the use of preventive measures. Based on a model of risk stratification done by an international TLS expert panel who made recommendations for TLS prophylaxis in 2010, frequent laboratory monitoring, aggressive intravenous hydration and a single dose of rasburicase (0.1-0.2 mg/kg) were recommended for patients with high risk for TLS (depending on the type of tumor; "high risk" includes: leukocytosis of more than 100*10^9/L, stage III/IV tumors, doubling of LDH level, or renal dysfunction with elevated potassium, phosphorus, and uric acid), if clinically necessary, rasburicase can be repeated [14]. Tumor lysis syndrome is a serious complication of this rare subtype of B-cell NHL. In one retrospective observational study of ten years, two of six patients diagnosed with plasmablastic lymphoma presented with spontaneous tumor lysis syndrome. Unfortunately, all patients presenting with spontaneous tumor lysis syndrome expired before receiving chemotherapy. These patients had a mean CD4 count of <200/mm3 [3] compared to our patient's CD4 count of 375/mm3 (see Table 1).

**Table 1:** Comparison between our patient and other PBL known characteristics PBL - plasmablastic lymphoma; TLS - tumor lysis syndrome

Characteristic	Our patient	Usual PBL patients [5]
Age	49 years old	Around 40 years old
Sex	Male	Male < female
Median CD4 count	356 cells/UI	180 cells/UI
Location	Gastrointestinal tract (GIT) “Lesser Sac”	Oral cavity most common, other include GIT, lymph nodes, visceral cranium, cervix, thorax skin, eyelid, and retroperitoneum
Presentation with TLS	Yes	One study reported 33%, 2/6 (rarely stated in literature) [3]
Histopathology and immunohistochemistry	Positive for CD45, CD4, CD138, CD79a, CD19, and negative for CD20	Usually CD20 negative (positive only in <2% of cases) and plasmacytic markers (CD38, CD79a, CD138, and/or MUM1, IRF4) positive
Epstein-Barr virus	Negative	Positive in > 80%

This description of cases emphasizes that PBL can present as TLS, and we can notice that the likelihood of TLS rises with elevated LDH, knowing it is a rapidly replicating tumor with high tumor burden. More future studies are needed to establish specific pathophysiology with this presentation. Spontaneous tumor lysis syndrome occurs rarely but has fatal consequences. Clinicians should be wary about the intrinsic and extrinsic risk factors with special attention to malignancies with a high proliferation rate [15].

## Conclusions

Plasmablastic lymphoma is a rare and aggressive HIV associated lymphoma. Spontaneous TLS can be a serious manifestation of PBL; as it is characterized by being a high-grade malignancy, and commonly presents as stage III/IV. We suggest that the clinicians should screen for the laboratory evidence of TLS when PBL is suspected and use guidelines for treatment as it could be a fatal emergency.
